# Mapping Preeclampsia Risk Thresholds to Screen-Positive Rates in Routine First-Trimester Screening: A Single-Centre Audit

**DOI:** 10.3390/diagnostics16203311

**Published:** 2026-10-12

**Authors:** Maria Cezara Mureșan, Marius Bogdan Mureșan, Dan Dumitrașcu Biriș, Ioan Cosmin Cîtu

**Affiliations:** 1 Department of Obstetrics and Gynecology, Faculty of Medicine, “Victor Babeș” University of Medicine and Pharmacy, 300041 Timișoara, Romania; citu.ioan@umft.ro; 2 Maternal-Fetal Medicine Center, 300331 Timișoara, Romania

**Keywords:** preeclampsia, first-trimester screening, Fetal Medicine Foundation algorithm, screen-positive rate, risk threshold, aspirin prophylaxis, implementation audit, ascertainment bias

## Abstract

**Background/Objectives:** First-trimester screening for preeclampsia (PE) with the Fetal Medicine Foundation (FMF) competing-risks algorithm assigns each pregnancy a numeric risk (1:X), which is compared against a threshold to decide aspirin eligibility. What proportion of a real-world screened population a given numeric threshold actually selects—its screen-positive rate (SPR)—depends on which markers were available and on the population’s underlying risk distribution, independently of how well that threshold detects PE. We audited this relationship directly from routine data from a single centre. **Methods:** Retrospective cohort of 5493 singleton pregnancies with a first-trimester PE risk calculated at 11–14 weeks (2020–2026) in one Romanian fetal-medicine centre. Pregnancies were stratified into five groups according to which markers were available at screening (maternal factors ± biochemistry [PAPP-A] ± uterine artery pulsatility index [UtA-PI] ± mean arterial pressure [MAP]), and the centre-specific SPR at fixed risk thresholds and the risk threshold corresponding to fixed SPR targets of 5%, 10%, 15% and 20% were computed for each group. The operational clinical threshold (1:150 before and 1:100 after the introduction of systematic MAP measurement in October 2023) was benchmarked against this map. Outcome ascertainment was quantified to determine whether these data could support any estimate of detection performance. **Results:** Among pregnancies screened with the maternal factors + MAP + UtA-PI + PAPP-A combination now used in routine practice (*n* = 1797), the operational threshold of 1:100 corresponded to an SPR of 16.7%, closer to the 15% than the 10% mapping point (local threshold for SPR = 10%: 1:66); the same numeric threshold corresponded to an SPR of only 11.0% among pregnancies screened with maternal factors and UtA-PI alone (*n* = 317). The historical reduction in SPR from 25.0% (pre-October 2023, threshold 1:150) to 16.1% (post-October 2023, threshold 1:100) was attributable mainly to the threshold change itself, since a same-era comparison at an identical fixed threshold (1:100) showed little difference in SPR between examinations with and without MAP (16.2% vs. 16.0%). Documented pregnancy outcome was available for only 13.5% of pregnancies (742/5493) and was strongly enriched for complicated pregnancies (documented PE prevalence 5.53% in this subset versus 0.87% in the whole cohort and 0.87–1.2% in published Romanian single-centre series). Consequently, outcome data in this dataset were considered unsuitable for local estimation of detection performance or treatment effect, and no such estimate is offered. **Conclusions:** The numeric risk threshold used clinically does not correspond to a fixed screen-positive rate across marker combinations in routine practice; local mapping of this relationship is a low-cost, outcome-independent audit that any screening centre can perform on its own data before, and independently of, any attempt to validate detection performance.

## 1. Introduction

Preeclampsia (PE) complicates 2–8% of pregnancies worldwide and remains a leading cause of maternal and perinatal morbidity and mortality [1,2]. First-trimester screening with the Fetal Medicine Foundation (FMF) competing-risks algorithm, which combines maternal characteristics with mean arterial pressure (MAP), uterine artery pulsatility index (UtA-PI) and placental biochemistry (placental growth factor [PlGF] and/or pregnancy-associated plasma protein-A [PAPP-A]), assigns each pregnancy a patient-specific risk of delivering with PE before a given gestational age [3,4,5,6,7,8]. Each of these markers has been shown individually to improve first-trimester prediction of PE beyond maternal factors alone, including mean arterial pressure [9,10], and biophysical and biochemical marker combinations more broadly [11,12]. In the ASPRE trial, women identified as high-risk by this algorithm and treated with 150 mg aspirin nightly from 11 to 14 weeks had a 62% reduction in preterm PE compared with placebo [13]. This benefit proved dependent on adherence and timing: the protective effect of aspirin in ASPRE was significantly greater among women who were compliant with the prescribed regimen [14], and pooled evidence indicates that aspirin’s efficacy against preterm PE is concentrated when treatment is initiated at or before 16 weeks’ gestation and given at a dose of at least 100 mg/day [15,16], observations that motivate explicit calls for timely clinical implementation of first-trimester screening-and-prevention programmes of the kind audited here [17]. This evidence underlies current recommendations from FIGO, ISUOG and national societies for first-trimester PE screening with risk-based aspirin prophylaxis [18,19].

A risk threshold—conventionally expressed as 1:X—must be chosen to decide which pregnancies are offered aspirin. Two related but distinct proportions are relevant to this choice, and the distinction matters for how audit findings such as ours should be read. The screen-positive rate (SPR) is the proportion of the whole screened population, irrespective of outcome, whose calculated risk exceeds the threshold; it is an outcome-independent, purely descriptive property of a population and a threshold, computable from screening data alone. The false-positive rate (FPR), by contrast, is conventionally reported alongside a detection rate (DR) as the proportion of women who do *not* subsequently develop PE who are nonetheless screen-positive—an outcome-dependent quantity that requires reliable ascertainment of who did and did not develop PE. Because PE is comparatively rare, SPR and FPR are numerically close in most populations, but they are not interchangeable, and published detection-rate tables (typically expressed “at FPR 10%”) describe operating points defined by outcome-verified performance, not by population screen-positive proportion alone. This distinction is central to how the present study should be interpreted: what we measure directly and exclusively is SPR; published DR-at-FPR figures are used only as external context for the general shape of algorithm performance, not as a conversion of our SPR-based thresholds into expected detection rates.

What is less widely appreciated in routine practice is that the same numeric risk threshold does not necessarily select the same screen-positive proportion of a population across different marker combinations or across different populations. Tan et al. demonstrated this directly with respect to population: applying an identical complete algorithm (including PlGF) at the fixed risk cutoff of 1:100 for preterm PE yielded a screen-positive rate of 10% in Caucasian women but 34% in women of Afro-Caribbean racial origin, reflecting a substantially higher background prevalence of preterm PE in the latter group [20]. An analogous phenomenon should be expected whenever the marker set available at screening changes—for instance, when PlGF is not measured, or when MAP is added to a protocol that previously relied on maternal factors, biochemistry and UtA-PI alone—because differences in marker availability may alter the distribution of calculated risks and therefore the fraction exceeding a given cutoff. To our knowledge, no study has directly quantified how a single centre’s operational threshold’s screen-positive rate shifts across the marker combinations that arise naturally within its own routine practice.

This question has direct practical relevance. If a centre’s operational threshold (say, 1:100) was adopted with the intention of flagging approximately 10% of the screened population, but in practice selects a substantially larger proportion because of the marker combination in routine use, then aspirin prophylaxis is being offered more broadly than intended. Broader eligibility increases the number of women exposed to prophylaxis and therefore changes the balance between expected case detection, treatment burden, adherence requirements and potential adverse effects—a programmatic consequence worth quantifying locally, independent of any claim about the biological efficacy of aspirin itself. Understanding the true population share selected by a given numeric threshold is therefore a low-cost, outcome-independent audit that any screening centre with a research database can perform on its own data.

Our centre introduced systematic maternal blood-pressure measurement (used to compute MAP) into first-trimester PE screening only from October 2023; before this date, most examinations combined maternal factors, biochemistry and UtA-PI without MAP. The clinical recommendation for aspirin prophylaxis was risk >1:150 before this change and >1:100 afterwards. This administrative history created, within a single centre’s own data, several naturally occurring marker-combination subgroups, allowing SPR mapping without needing to reconstruct or re-implement the proprietary FMF algorithm.

We therefore performed an internal calibration audit with two deliberately outcome-independent aims: (i) to quantify, for each marker combination used in routine first-trimester PE screening at our centre, the screen-positive rate at the operational clinical threshold and the local threshold corresponding to fixed SPR targets of 5%, 10%, 15% and 20%; and (ii) to characterise, transparently and quantitatively the completeness and potential bias of routinely available pregnancy-outcome data at our centre in order to establish—rather than assume—whether these data could support any further inference about detection performance or treatment effect, as well as to justify why such inference is deferred to subsequent work on this same cohort once outcome ascertainment has been actively strengthened.

## 2. Materials and Methods

### 2.1. Study Design and Setting

This was a retrospective cohort study of prospectively recorded routine clinical data from the Maternal-Fetal Medicine Center, Timișoara, a private fetal-medicine unit in western Romania performing first-trimester combined screening for a largely self-referred and physician-referred population. All examinations are recorded in a structured obstetric ultrasound database (Astraia, Astraia Software GmbH, Munich, Germany), including maternal characteristics, biometry, biochemical analytes and their multiples of the median (MoM), uterine artery Doppler, blood pressure measurements, the computed FMF preeclampsia risk, and, when available, pregnancy outcome. This study is reported in line with the STROBE recommendations for cohort studies.

### 2.2. First-Trimester PE Risk Calculation and Data Source

Throughout the study period, first-trimester PE risk was computed within the Astraia module from whichever combination of maternal factors, biochemistry and biophysical markers was available at the time of the examination: maternal factors were always included; PAPP-A (reported as MoM) was included when biochemical screening had been performed; UtA-PI (mean of left and right pulsatility index, MoM) was included when Doppler assessment had been performed, following the standard FMF measurement protocol [21]; and MAP, computed from two measurements on each arm using the FMF-recommended protocol [22], was included from the point at which blood-pressure measurement was systematically incorporated into the protocol (October 2023 onward; sporadic measurement occurred earlier). Free β-human chorionic gonadotropin (β-hCG), also recorded in the database, is not an input of the FMF PE-risk calculator and was not considered a PE marker in this analysis [4]. Placental growth factor (PlGF) was not measured at our centre during the study period and was therefore never a component of the risk calculated here; this marker set is accordingly referred to throughout as the “maternal factors + MAP + UtA-PI + PAPP-A combination,” not as a complete or maximal implementation of the FMF algorithm, since higher-performing combinations that include PlGF have been reported elsewhere (Section 2.5). The computed risk is expressed by the software as a fraction 1:X (X = risk denominator), stored numerically and used directly as our variable of interest (“risk”). The recommendation for aspirin prophylaxis (150 mg nightly, from before 16 weeks) was a risk greater than 1:150 before the introduction of systematic MAP measurement and a risk greater than 1:100 thereafter, following the centre’s internal protocol update; this operational recommendation, not any single marker set, is the threshold audited here.

The source file Risc PE la T1.xls is a direct export of the Astraia “PE Risks” module (65,087 examination rows, 2009–2026). Patient name fields were removed before any aggregate computation; no row-level data were retained outside the secure analysis environment.

### 2.3. Study Population

The index examination for each pregnancy was the first examination performed in the “12-week” screening window with a numeric PE risk >0. Multiple pregnancies (more than one fetus, or recorded chorionicity) were excluded, and crown–rump length was required to be 45–84 mm, consistent with first-trimester combined screening criteria. When more than one qualifying examination existed for the same pregnancy (identified by patient ID and case number), the earliest was retained. This yielded 5493 singleton pregnancies, 2020–2026.

### 2.4. Marker-Combination Groups and Local Screen-Positive-Rate Mapping

For each pregnancy, presence or absence of PAPP-A, UtA-PI and MAP at the index examination was recorded from the corresponding database fields (maternal factors were present in all examinations by definition). Five marker-combination groups accounting for 98.3% of the cohort were defined a priori from the data, without re-implementing the FMF algorithm: (i) maternal factors + biochemistry + UtA-PI (no MAP); (ii) maternal factors + biochemistry + UtA-PI + MAP; (iii) maternal factors + biochemistry only; (iv) maternal factors + UtA-PI + MAP (no biochemistry); and (v) maternal factors + UtA-PI only (no biochemistry, no MAP). These are naturally occurring, largely non-overlapping subgroups of different pregnancies whose available markers differed—not the same pregnancies with risk recalculated under different marker subsets—so any association between marker combination and SPR reflects the joint influence of marker availability and of whichever other characteristics differ between these subgroups (Section 4.2 and Section 4.6 discuss this explicitly).

Within each group and for the cohort as a whole, the screen-positive rate (SPR)—the outcome-independent proportion of the group whose calculated risk exceeded a given threshold—was computed at the fixed clinical threshold of 1:100. Separately, the risk denominator (X in “1:X”) corresponding to fixed SPR targets of 5%, 10%, 15% and 20% of each group was computed (numpy.percentile, linear interpolation). No outcome data were used in this analysis; SPR and the local threshold map are properties of the screened population and the risk distribution alone.

### 2.5. Literature Context for Algorithm Performance

Because reproducing a full detection-rate curve for our exact marker combination (maternal factors + MAP + UtA-PI + PAPP-A, without PlGF or β-hCG) would require prospective, complete PE-outcome ascertainment unavailable in this dataset (Section 2.6), published detection-rate figures are used here only as external, outcome-dependent context for the general performance of related marker combinations—not as a means of converting our locally observed SPR into an expected detection rate. Two primary sources were used, verified directly from the original publications: a Romanian cohort study reporting DR at FPR 10% for exactly our marker combination [23], and the classical multi-combination table of Tan et al., which additionally reported an SPR comparison across two ethnic subgroups using an identical fixed algorithm, providing independent, outcome-verified evidence for the population dependence of a fixed numeric threshold that is conceptually analogous to, but methodologically distinct from, our own SPR mapping across marker combinations [20].

### 2.6. Outcome Ascertainment: A Deliberately Limited, Justificatory Analysis

Pregnancy outcome in this database is entered from the referring physician, the delivery unit or the patient and is known a priori to be incompletely captured because many pregnancies deliver at institutions that do not report back to the centre. The purpose of the analysis in this section is narrow and specific: to establish quantitatively rather than by assumption whether outcome data in the present dataset could support any local estimate of detection performance or treatment effect, or, if not, to characterise the bias precisely enough to justify deferring such estimates to future work on this cohort, rather than to serve as a parallel results stream in its own right.

Every examination row associated with each pregnancy (patient ID and case number), not only the index first-trimester examination, was searched for any documented delivery-related field (mode of delivery, gestational age at delivery, birth weight, or pregnancy evolution [live birth/miscarriage/intrauterine death/termination]). A pregnancy was classified as “outcome-ascertainable” if at least one such field was recorded on any visit. Documented PE was defined as a positive value in either of two independent PE-outcome fields present in the database (these two fields never co-occurred within the same pregnancy, consistent with them originating from different sections of the clinical report, and were therefore combined with a logical OR). PE diagnoses were not independently adjudicated against standardised diagnostic criteria by the study team; they represent diagnoses as documented in the routine clinical record by the managing physician, delivery unit or referring service. Gestational age at PE diagnosis or delivery was insufficiently and selectively ascertained in this dataset (Section 3.6) to permit a reliable early- versus late-onset classification, or an analysis of the association between marker combination and PE timing; both questions are deferred to the actively ongoing outcome follow-up for this cohort (Section 4.6).

Two descriptive analyses were performed. First, documented PE prevalence in the whole cohort versus in the outcome-ascertainable subset was compared to characterise the direction and likely mechanism of ascertainment bias. Second, as a strictly exploratory, hypothesis-generating observation reported in the Supplementary Material rather than in the principal results, we determined, for each of the 48 pregnancies with documented PE, whether the first-trimester risk had exceeded the threshold in clinical use at the time of screening. This case-capture proportion is explicitly not presented as a sensitivity or detection-rate estimate: the documented-PE subset is itself subject to the ascertainment bias quantified above, and aspirin prescribed to flagged pregnancies may alter whether and how PE subsequently presents, confounding any attempt to interpret the figure as unmodified screening performance. It is reported in the Supplementary Material precisely because, despite this caveat being stated explicitly, a percentage this close to a familiar sensitivity figure risks being read as one; the same caveat applies to a pre-specified comparison of documented PE prevalence before and after the October 2023 protocol change, restricted to the ascertainable subset, also reported in the Supplementary Material only, because ascertainment completeness itself differed materially between the two periods (Section 3.6), precluding a straightforward before/after interpretation.

### 2.7. Statistical Analysis

Proportions are reported with Wilson 95% confidence intervals. Percentile-based thresholds were computed empirically per group as described above; no parametric distributional assumption was applied to the risk variable. Baseline cohort characteristics (Section 3.2) were summarised descriptively for the whole cohort—median [interquartile range, IQR] for continuous variables, *n* (%) for categorical variables—without formal between-group hypothesis testing; maternal height and weight values outside physiologically plausible ranges (140–200 cm; 35–160 kg) were treated as implausible data-entry values and were excluded from the body mass index calculation only. All between-group comparisons of the screen-positive rate across marker combinations reported in this audit are descriptive and associational; no causal effect of adding or removing an individual marker is inferred (Section 2.4 and Section 4.3). Analyses were performed in Python 3.11 (pandas 2.2, numpy 1.26).

### 2.8. Ethics

This study was conducted in accordance with the Declaration of Helsinki and was approved by the Ethics Committee of the Maternal-Fetal Medicine Center, Timișoara, Romania (initial approval No. 1/2026, 1 August 2026; framework approval No. 2/2026, 7 September 2026, as amended by Addendum No. 2, covering all retrospective secondary analyses of the database). Patient consent was waived because this study is a retrospective, non-interventional analysis of pseudonymised routine clinical data; only aggregate results are reported.

## 3. Results

### 3.1. Study Population and Historical Context

Of 5493 singleton pregnancies with a first-trimester PE risk (2020–2026), systematic MAP measurement was essentially absent before September 2023 (<2% of examinations) and became near-universal from 2024 onward (86–92% of examinations per year), with a transitional quarter (October–December 2023) rising from 19% to 88%. Under the period-appropriate operational threshold (1:150 before and 1:100 after the protocol change), SPR fell from 25.0% pre-change to 16.1% post-change—a relative reduction of about 36%. A same-era internal comparison at the identical fixed threshold (1:100), however, showed the presence or absence of MAP made little difference to SPR (16.2% with MAP vs. 16.0% without, among examinations performed in the same period). This indicates that the historical reduction in SPR reflects mainly the threshold tightening (1:150→1:100) rather than a large shift in the risk distribution attributable to MAP itself; whether MAP nonetheless improves the detection of true PE cases at a matched SPR is a distinct, outcome-dependent question that this outcome-independent audit cannot address.

### 3.2. Cohort Characteristics

The baseline characteristics of the whole screened cohort are summarised in Table 1, reported descriptively for the cohort as a whole rather than stratified by marker-combination group (Section 2.7).

### 3.3. Marker-Combination Groups

Five marker-combination groups, representing distinct subgroups of pregnancies rather than the same pregnancies recalculated under different marker subsets, accounted for 5397/5493 pregnancies (98.3%) (Table 2).

### 3.4. Local Screen-Positive-Rate Mapping

The principal finding of this audit is that a fixed numerical risk threshold did not select a fixed proportion of pregnancies across the marker combinations encountered in routine practice. Table 3 shows the risk denominator corresponding to the 5th, 10th, 15th and 20th SPR target within each marker-combination group, alongside the SPR each group actually achieved at the fixed clinical threshold of 1:100. The local threshold mapping and the corresponding screen-positive rates are illustrated in Figure 1 and Figure 2, respectively. All figures in this section are outcome-independent SPR values, not FPR or detection-rate estimates.

For the maternal factors + MAP + UtA-PI + PAPP-A combination—the configuration now used in routine practice—the operational threshold of 1:100 corresponded to an SPR of 16.7%, closer to the 15% than the 10% mapping point; the local threshold at which SPR = 10% in this group was 1:66. The same fixed numeric threshold (1:100) corresponded to an SPR of only 11.0% among pregnancies screened with maternal factors and UtA-PI alone. In practical terms, at a fixed 1:100 threshold, approximately 167 of every 1000 women screened with the current maternal factors + MAP + UtA-PI + PAPP-A configuration would be classified as aspirin-eligible, compared with approximately 110 per 1000 among those screened with maternal factors and UtA-PI alone—a difference of about 57 additional women per 1000 screened. This comparison describes a difference in treatment eligibility, not in additional PE cases detected, which cannot be estimated from the present outcome data (Section 3.6). SPR at a fixed numeric threshold therefore varied appreciably (11.0–16.7%) across the marker combinations present in routine practice at this centre; this variation is associated with, rather than demonstrated to be caused by, the marker combination available at screening (Section 4.2).

### 3.5. Context from Published Detection-Rate Studies

A Romanian cohort using exactly our marker combination (maternal factors + MAP + UtA-PI + PAPP-A, without PlGF or β-hCG; 1372 pregnancies after excluding 211 that had received aspirin; 12 PE cases) reported a detection rate of 71.4% at a fixed false-positive rate of 10% (area under the ROC curve 0.929), compared with 50.0% for maternal factors alone (AUC 0.841) [23]. At their own operational threshold of 1:100, sensitivity was 71.4%, specificity 91.7% and their outcome-verified screen-positive rate 8.8%—numerically lower than the 16.7% SPR we observed for the equivalent marker combination and threshold in our own cohort. Because their 8.8% is an outcome-verified figure at a threshold their cohort’s own data happened to place near FPR 10%, while our 16.7% is a purely population-based SPR at the same numeric threshold in a different cohort, this comparison illustrates cohort-to-cohort variability rather than a discrepancy in algorithm performance, and should not be read as evidence that our threshold under- or over-detects PE, which we did not assess.

The classical multi-combination table of Tan et al., using the complete algorithm including PlGF (not measured at our centre), reported detection rates at a fixed 10% false-positive rate ranging from 52.6% (maternal factors alone) to 89.7% (maternal factors + MAP + UtA-PI + PlGF) for early PE, with intermediate combinations (+MAP: 61.2%; +UtA-PI: 69.8%; +PlGF: 72.4%) bracketing the 71.4% reported for our marker combination [20,23]. The same study independently demonstrated the population dependence of a fixed numeric threshold using outcome-verified data: applying the identical complete algorithm at the fixed risk cutoff of 1:100 for preterm PE gave a screen-positive rate of 10% in Caucasian women but 34% in women of Afro-Caribbean racial origin (detection rates for early, preterm and term PE of 88%/69%/40% and 100%/92%/75%, respectively), reflecting the higher background prevalence of preterm PE in the latter group rather than a difference in the fixed cutoff itself [20]. This finding is conceptually analogous to, though methodologically distinct from (it is outcome-verified SPR-vs-FPR variation by population, whereas our finding is purely population-based SPR variation by marker combination), the pattern we observed across marker combinations within a single population (Section 3.4): a fixed numeric threshold does not guarantee a fixed screen-positive rate whenever the underlying risk distribution differs, whether because of population characteristics or because of the marker set applied.

### 3.6. Outcome Ascertainment

Any documented pregnancy outcome (mode of delivery, gestational age at delivery, birth weight, or pregnancy evolution) was available for only 742/5493 pregnancies (13.5%, 95% CI 12.6–14.4%); the remaining 86.5% had no outcome documentation in this database. Documented PE (either of two independent outcome fields, which never co-occurred) was recorded in 48/5493 pregnancies overall (0.87%, 95% CI 0.66–1.16%), but in 41/742 (5.53%, 95% CI 4.10–7.41%) of the outcome-ascertainable subset—four- to six-fold higher than both the crude cohort figure and published Romanian single-centre estimates of PE incidence (0.87% [23] and 1.2% [24], both single-centre, unselected obstetric populations; Table 4). This pattern indicates that outcome ascertainment at our centre is not missing at random but is enriched for complicated pregnancies, most plausibly because women with an uncomplicated course more often deliver, and are not subsequently followed, at institutions that do not report back into this database. The enrichment of documented preeclampsia among pregnancies with ascertainable outcomes is illustrated in Figure 3. As a secondary methodological finding, this result demonstrates why the present dataset cannot support a local estimate of PE incidence, screening sensitivity, or treatment effect, and why such estimates are deferred to future work with actively strengthened outcome ascertainment rather than attempted here.

A pre-specified sensitivity comparison of documented PE prevalence before and after the October 2023 protocol change, restricted to the outcome-ascertainable subset, is reported in the Supplementary Material rather than in the principal results: outcome ascertainment completeness itself differed materially between the two periods (16.0% of pre-change pregnancies were ascertainable versus 10.4% post-change), confounding any before/after comparison with a temporal maturation effect unrelated to screening or treatment.

The exploratory case-capture observation among the documented PE cases and its full results table are reported in the Supplementary Material (Table S2) rather than here to avoid a descriptive percentage that superficially resembles a sensitivity figure being read as one alongside this study’s principal, outcome-independent findings (see Section 4.5).

## 4. Discussion

### 4.1. Principal Findings

In 5493 first-trimester PE risk assessments from a single Romanian fetal-medicine centre, the numeric risk threshold used to trigger aspirin prophylaxis did not correspond to a fixed screen-positive rate across marker combinations in routine use: the operational threshold of 1:100, applied to the maternal factors + MAP + UtA-PI + PAPP-A combination now used in routine practice, corresponded to an SPR of 16.7%—closer to a 15% than a 10% screen-positive rate—while the identical numeric threshold corresponded to an SPR of only 11.0% among pregnancies screened with maternal factors and UtA-PI alone. An outcome-verified finding from the literature using a different mechanism (population ethnicity rather than marker availability) but demonstrating the same underlying principle—a fixed complete algorithm applied at the same risk cutoff (1:100) selecting an SPR of 10% in Caucasian women but 34% in women of Afro-Caribbean racial origin [20]—supports the general proposition that a fixed numeric threshold’s screen-positive rate is not transportable across populations with different underlying risk distributions, of which our marker-combination finding is a population-based, outcome-independent, single-centre instance. Separately, we quantified rather than assumed the well-known limitation of routine-practice outcome data in a referral fetal-medicine setting: only 13.5% of pregnancies had any documented delivery outcome, and this subset was itself enriched for pathology (PE prevalence 5.53% versus 0.87–1.2% in comparable published cohorts). This finding is reported as a secondary methodological result establishing why local validation of screening performance and treatment effect is deferred to subsequent work on this cohort, not as a parallel set of clinical conclusions to be drawn from the present, outcome-independent audit.

### 4.2. Screen-Positive Rate Is Not the Same Quantity as False-Positive Rate

A central methodological point of this study, stated explicitly here to avoid a common source of confusion when interpreting threshold-mapping exercises, is that everything we report about SPR is outcome-independent and describes population selection by a threshold, not detection performance. Published figures expressed “at FPR 10%” describe an outcome-verified operating point—the proportion of non-PE pregnancies that screen positive—which is numerically close to, but conceptually and, in finite samples, numerically distinct from, SPR, the proportion of the whole population that screens positive irrespective of outcome. Our finding that the maternal factors + MAP + UtA-PI + PAPP-A combination’s local threshold for SPR = 10% is 1:66 should not be read as implying that this threshold operates at FPR = 10% in the sense of published detection-rate tables; no claim about detection rate at this or any other threshold is made in this study because our data cannot support one (Section 3.6 and Section 4.6). The value of the SPR mapping in Table 3 is precisely that it can be computed and used to inform threshold-setting policy, entirely independently of outcome ascertainment—a property we consider a strength rather than a limitation of the present analysis, and the basis for treating this as a distinct, self-contained first study rather than a preliminary or partial validation.

### 4.3. Why the Numeric Threshold and the Screen-Positive Rate Diverge Across Marker Combinations

The FMF competing-risks model estimates each pregnancy’s individual risk from a modelled distribution of gestational age at PE onset, conditioned on maternal characteristics and biomarker values; the population SPR at any numeric cutoff is therefore the proportion of that population’s aggregate risk distribution beyond the cutoff, not a fixed property of the cutoff itself [3,4,8]. Differences in marker availability may alter the distribution of calculated risk; however, the direction and magnitude cannot be attributed to individual markers in the present design. Our data show this pattern descriptively: the group screened with maternal factors and UtA-PI alone required the loosest threshold (1:63) to flag its top 5%, compared with 1:41 in the maternal factors + MAP + UtA-PI + PAPP-A group. However, because our five marker-combination groups are naturally occurring subgroups of different pregnancies—defined by which markers happened to be recorded—rather than the same pregnancies with risk recalculated under successively added or removed markers, we can state only that SPR differed across pregnancies screened with different marker combinations, not that adding or removing any specific marker caused this difference. Marker availability at our centre is also partially confounded with calendar period (MAP became systematic only from October 2023, and biochemistry or Doppler availability may itself have shifted over the study period for reasons unrelated to marker choice, such as changes in referral mix or laboratory logistics), so period-related population differences cannot be fully separated from marker-combination effects with the present design. A within-pregnancy design—recalculating risk for the same pregnancies with and without each marker—would be needed to support a causal claim, and is outside the scope of this outcome-independent audit.

### 4.4. Context from Published Detection Rates

Our closest literature comparator, using our exact marker combination, reported a detection rate of 71.4% at a fixed 10% false-positive rate in a Romanian cohort of 1372 pregnancies [23]—plausibly consistent with the position of our marker combination’s reported detection rate between the “+UtA-PI” and “+PlGF” rows of the more extensively characterised Tan et al. table [20], though this is an indirect, cross-cohort, outcome-dependent comparison and not evidence from our own data, which contain no outcome-verified detection-rate estimate. A routine-practice audit from Hungary, conducted, as ours was, within the Astraia platform, similarly reported real-world performance of first-trimester PE screening outside the original FMF development and validation cohorts, reinforcing that our own centre’s experience sits within a broader pattern of consistent, if numerically variable, performance when the algorithm is applied in everyday clinical practice rather than in a research setting [25].

The competing-risks model itself has been externally validated, with good calibration and discrimination, in cohorts distinct from those used for its original development [26,27,28], and this transportability has since been confirmed more broadly: a systematic review and meta-analysis of externally validated preeclampsia prediction models found the FMF competing-risks algorithm to be the most consistently validated model across settings, with a pooled area under the curve of approximately 0.90 for preterm PE despite this heterogeneity in implementation [29]. Other external-validation cohorts have, however, reported more modest performance when the algorithm was applied outside its development population [30,31], underscoring that transportability is not guaranteed and that, as we argue throughout this study, local calibration—of which our own SPR mapping is one deliberately outcome-independent form—remains necessary even for an algorithm with strong average external validity.

A further, pragmatic source of cross-study variability is which biochemical marker is used: PAPP-A, measured at our centre, and PlGF are not interchangeable inputs, and a routine-practice comparison of the two found that switching from PAPP-A to PlGF materially changed screen-positive and detection performance despite both being valid FMF-algorithm inputs, illustrating that marker availability—not only marker choice in principle—shapes what a given centre’s screening programme actually achieves [32]. This observation parallels our own finding that marker-combination availability shapes the local screen-positive rate (Section 3.4 and Section 3.5): PAPP-A/PlGF choice and MAP/UtA-PI/biochemistry availability are both examples of how routine, non-protocol-driven variation in marker sets can shift what a fixed numeric threshold delivers in practice, quite apart from any change in the underlying algorithm itself. We report this only as context for the plausibility of the marker combination’s general performance ceiling and for the transportability of the underlying model, not as a claim about how our own threshold performs.

### 4.5. The Ascertainment-Bias Finding as a Secondary Methodological Result

An earlier iteration of this study proposed comparing observed PE incidence in our aspirin-eligible population against national or single-centre Romanian incidence figures as a signal of a possible therapeutic effect. On critical appraisal, and as directly demonstrated by restricting the denominator to pregnancies with any documented outcome (Section 3.6), this comparison is not supportable: rather than converging toward external benchmarks as ascertainment improved, PE prevalence within the outcome-ascertainable subset rose to four- to six-fold above them, indicating that pregnancies re-entering the database with a documented outcome are systematically enriched for complications rather than missing at random. We report the magnitude and direction of this bias as a secondary, quantified methodological finding—useful to other referral-based fetal-medicine registries with similarly incomplete back-reporting from delivery units—rather than as a co-equal analytic thread of the present study. The exploratory case-capture observation (Supplementary Table S2) is presented strictly as a descriptive characterisation, not as a sensitivity estimate or a principal finding of this audit.

### 4.6. Strengths and Limitations

Strengths include the use of naturally occurring marker-combination subgroups arising from routine documentation completeness, avoiding the need to re-implement a proprietary risk algorithm; a design that is entirely outcome-independent for its principal aim, and therefore immune to the ascertainment limitations that affect most single-centre screening audits; and a quantitative, rather than assumed, characterisation of outcome-ascertainment bias, obtained by exhaustively searching all recorded visits per pregnancy rather than relying on the index examination alone.

Limitations are nonetheless substantial. The five marker-combination groups are naturally occurring subgroups of different pregnancies rather than the same pregnancies with risk recalculated under different marker sets, so the association between marker combination and SPR cannot be interpreted causally, and marker availability is partially confounded with calendar period, as discussed in Section 4.3. This study makes no claim about detection rate, sensitivity, or treatment effect: outcome ascertainment was available for only 13.5% of the cohort and, as shown, biased toward complicated pregnancies, and no such estimate is offered as a result of this work. Medication documented at the first-trimester visit reflects pre-screening, physician-initiated aspirin rather than post-screening compliance with the risk-based recommendation, which is not re-documented after the initial visit; no analysis in this study attempts to estimate aspirin’s therapeutic effect. Placental growth factor was not measured at our centre, limiting direct comparability with the highest-performing marker combinations reported in the literature. The exact clinical decision rule (1:150 versus 1:100) was applied retrospectively from documented protocol history rather than extracted from an audit trail of individual clinical decisions. Finally, as in any single-centre audit, the generalisability of the specific numeric thresholds reported here to other populations or marker implementations is limited; the generalisable contribution is the demonstration that SPR-to-threshold mapping should be performed locally rather than assumed. Active outcome ascertainment for this cohort is ongoing and will enable future outcome-validated assessment of screening performance.

## 5. Conclusions

In a single-centre first-trimester preeclampsia screening cohort of 5493 singleton pregnancies, the numeric risk threshold used to guide aspirin prophylaxis corresponded to a materially different screen-positive rate depending on which markers were available at screening—16.7% for the maternal factors + MAP + UtA-PI + PAPP-A combination now in routine use at the operational 1:100 threshold (local SPR = 10% threshold: 1:66) versus 11.0% for maternal factors and UtA-PI alone—a pattern conceptually supported by published, outcome-verified evidence that a fixed algorithm’s screen-positive rate similarly varies with population risk distribution. This mapping is deliberately outcome-independent and therefore unaffected by the substantial ascertainment limitations we separately quantified: only 13.5% of the cohort had any documented delivery outcome, and this subset was itself enriched for pathology, precluding any local estimate of detection performance or treatment effect from the present data. Numeric screening thresholds intended to target a specific population screen-positive rate should be locally mapped against the marker combinations actually used in routine practice; this study establishes that mapping for one centre. Active outcome ascertainment for this cohort is ongoing and is expected to enable future outcome-validated assessment of screening performance.

## Figures and Tables

**Figure 1:**
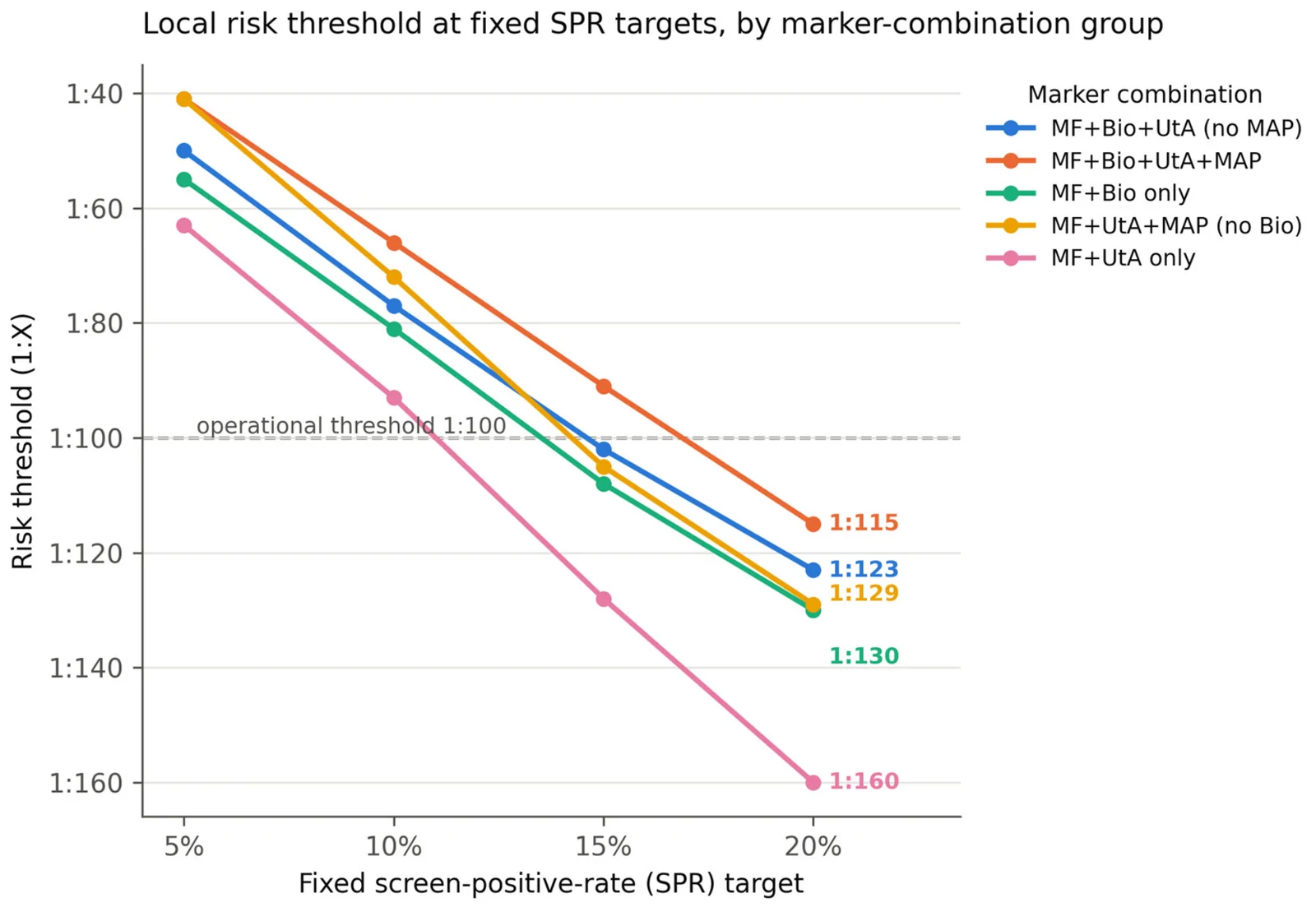
Local risk threshold (1:X, y-axis, inverted so that higher risk is upward) at fixed SPR targets of 5%, 10%, 15% and 20%, by marker-combination group; the horizontal reference line marks the operational clinical threshold of 1:100, with the SPR it produces in each group annotated. SPR, screen-positive rate.

**Figure 2:**
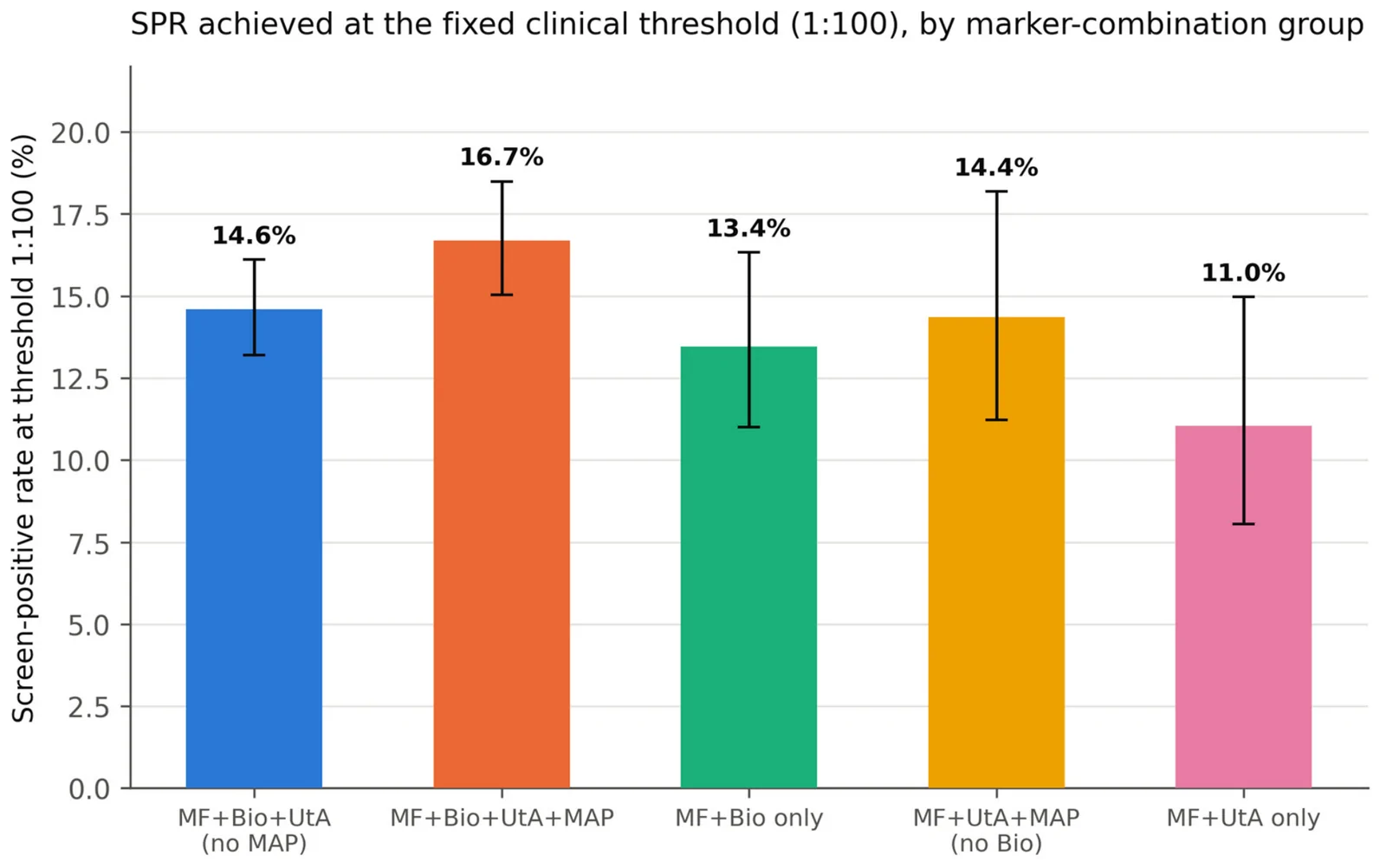
SPR achieved by each marker-combination group at the fixed clinical threshold of 1:100 (bars, exact 95% CI), ordered by marker content. SPR, screen-positive rate.

**Figure 3:**
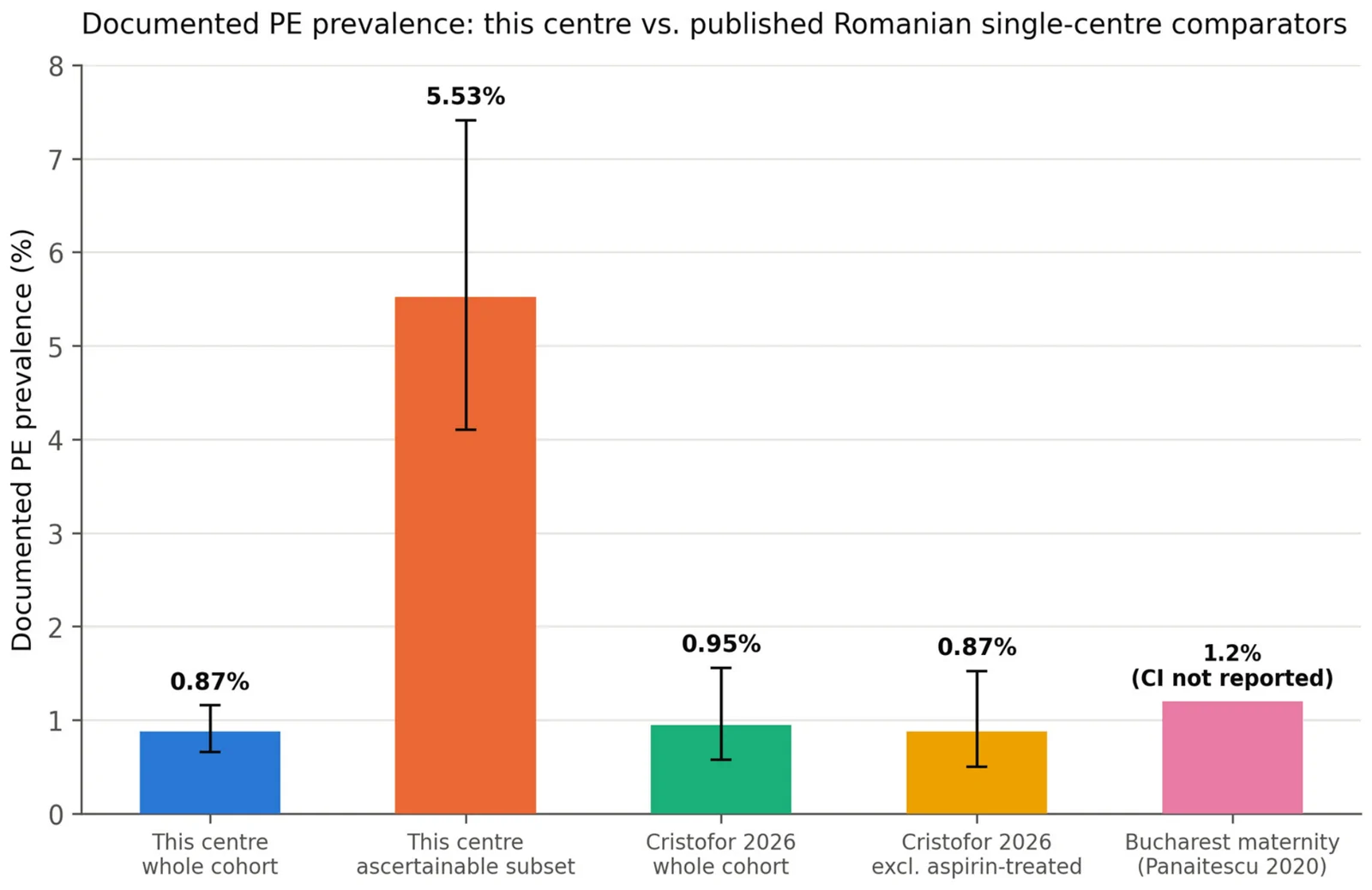
Documented PE prevalence: Crude cohort, outcome-ascertainable subset, and published Romanian single-centre comparators (bars, exact 95% CI where available), illustrating ascertainment enrichment for pathology [23,24].

**Table 1:** Baseline characteristics of the study cohort (*n* = 5493). Continuous variables are reported as median [IQR]; categorical variables as *n* (%). CRL, crown–rump length; BMI, body mass index; IQR, interquartile range.

Characteristic	Value
Maternal age (years)	31.5 [27.9–35.3]
BMI (kg/m^2^) ^a^	23.4 [21.0–27.2] (*n* = 5480)
Nulliparous	3492 (63.6%)
Current smoker ^b^	879/5491 (16.0%)
Conception by assisted reproduction (IVF/IUI/ovulation induction) ^b^	379/5492 (6.9%)
Chronic hypertension ^b^	73/5492 (1.3%)
Pre-existing diabetes (type 1 or 2)	38/5493 (0.7%)
Previous PE (≥24 weeks) among parous women ^c^	67/1998 (3.4%)
CRL at screening (mm)	65.9 [60.0–72.3]

^a^ 13 pregnancies (0.2%) excluded from the BMI calculation only due to height or weight values outside physiologically plausible ranges (140–200 cm; 35–160 kg), most likely data-entry errors. ^b^ Denominator reduced by missing data (<0.1% of the cohort). ^c^ Among the 2001 parous women (parity ≥ 1) in the cohort, with 3 missing values; not applicable to nulliparous women. Ethnicity was not systematically recorded in the clinical database and is not reported; the screened population is drawn predominantly from the Timișoara catchment area in western Romania.

**Table 2:** Marker-combination groups in the study cohort. Abbreviations: MF, maternal factors (present in every examination); Bio, biochemistry (PAPP-A); UtA, uterine artery pulsatility index; MAP, mean arterial pressure.

Marker Combination	*n*
MF + Bio + UtA (no MAP)	2261
MF + Bio + UtA + MAP	1797
MF + Bio only	632
MF + UtA + MAP (no Bio)	390
MF + UtA only	317

**Table 3:** Local risk threshold (1:X) at fixed SPR targets, as well as SPR achieved at the fixed clinical threshold 1:100, per marker-combination group. Abbreviations as in Table 2.

Group	*n*	1:X @5%	1:X @10%	1:X @15%	1:X @20%	SPR @1:100
MF + Bio + UtA (no MAP)	2261	1:50	1:77	1:102	1:123	14.6%
MF + Bio + UtA + MAP	1797	1:41	1:66	1:91	1:115	16.7%
MF + Bio only	632	1:55	1:81	1:108	1:130	13.4%
MF + UtA + MAP (no Bio)	390	1:41	1:72	1:105	1:129	14.4%
MF + UtA only	317	1:63	1:93	1:128	1:160	11.0%
Whole cohort	5493	1:46	1:74	1:101	1:125	14.9%

**Table 4:** Documented PE prevalence: Our centre versus published Romanian single-centre estimates.

Source	PE Prevalence	Population
This centre—documented prevalence, whole cohort	0.87% (48/5493)	crude, under-ascertained
This centre—outcome-ascertainable subset	5.53% (41/742)	enriched for complications
Cristofor et al. 2026, whole cohort [23]	0.95% (15/1583)	single-centre
Cristofor et al. 2026, after excluding aspirin-treated [23]	0.87% (12/1372)	single-centre
Large Bucharest maternity hospital, retrospective [24]	1.2%	single-centre, general population

## Data Availability

The pseudonymised dataset is held by the Maternal-Fetal Medicine Center, Timișoara, and is not publicly available because of patient-confidentiality restrictions; aggregate data are available from the corresponding author on reasonable request.

## Supplementary Materials

The following supporting information can be downloaded at https://www.mdpi.com/article/10.3390/diagnostics16203311/s1, Table S1: Pre- versus post-October 2023 documented PE prevalence within the outcome-ascertainable subset, with period-specific ascertainment rates (exploratory, confounded by differential ascertainment maturity between periods; not a principal result). Table S2: Case-capture at the T1 screening threshold among documented PE cases (*n* = 48), reported exploratory and hypothesis-generating only, not as a sensitivity or detection-rate estimate (Section 2.6). Among the 48 pregnancies with documented PE, 52.1% (25/48, 95% CI 38.3–65.5%) had already been flagged as high-risk at the threshold in clinical use at the time of screening (fixed 1:100); 54.2% (26/48, 95% CI 40.3–67.4%) at a fixed 1:150 threshold; and 52.1% (25/48, 95% CI 38.3–65.5%) under the period-appropriate threshold (1:150 pre- and 1:100 post-October 2023).
