# Supplementary material for: Mapping Preeclampsia Risk Thresholds to Screen-Positive Rates in Routine First-Trimester Screening: A Single-Centre Audit
Source: Diagnostics (Basel). 2026 Oct 12;16(20):3311. doi: 10.3390/diagnostics16203311 (PMC13703336; doi:10.3390/diagnostics16203311)
Supplement: Supplementary file 1 [file diagnostics-16-03311-s001.zip › diagnostics-4590888-supplementary.pdf]

Supplementary Materials

Mapping Preeclampsia Risk Thresholds to Screen-Positive Rates in Routine First-Trimester Screening: A Single-Centre Audit

Maria Cezara Mureșan, Marius Bogdan Mureșan, Dan Dumitrașcu Biriș and Ioan Cosmin Cîtu

---

Table S1. Pre- versus post-October 2023 documented PE prevalence within the outcome-ascertainable subset. This comparison is exploratory and is not presented as a principal result: outcome-ascertainment completeness itself differed materially between the two periods, confounding any before/after interpretation with a temporal maturation effect unrelated to screening or treatment (see manuscript Section 3.6).

Period	Outcome-ascertainable / total pregnancies	Ascertainment rate	Documented PE / ascertainable	PE prevalence (95% CI)
Pre-October 2023 (threshold 1:150)	488	16.0%	21/488	4.30% (2.83–6.49%)
Post-October 2023 (threshold 1:100)	254	10.4%	20/254	7.87% (5.15–11.85%)

Note: Confidence intervals are Wilson 95% CIs. The apparent rise in documented PE prevalence after October 2023 runs in the opposite direction to what a protective effect of the tightened threshold (or of systematic MAP measurement) would predict, but the confidence intervals overlap substantially and ascertainment completeness itself fell across the same period (16.0% to 10.4%), so no causal or protective interpretation is drawn from this comparison.

Table S2. Case-capture at the T1 screening threshold among documented PE cases (n = 48), reported as an exploratory, hypothesis-generating observation only. This proportion is explicitly not a sensitivity or detection-rate estimate: the documented-PE denominator is drawn from the outcome-ascertainable subset already shown to be enriched for complications (manuscript Section 3.6), and aspirin prescribed to flagged pregnancies may itself modify whether and how PE subsequently presents and is documented (manuscript Section 2.6).

Threshold applied	Flagged / documented PE cases	% (95% CI)
Fixed 1:100	25/48	52.1% (38.3–65.5%)
Fixed 1:150	26/48	54.2% (40.3–67.4%)
Period-appropriate (1:150 pre-, 1:100 post-October 2023)	25/48	52.1% (38.3–65.5%)
— of which pre-October 2023 subgroup (threshold 1:150)	8/21	38.1% (20.8–59.1%)
— of which post-October 2023 subgroup (threshold 1:100)	17/27	63.0% (44.2–78.5%)

Note: Confidence intervals are Wilson 95% CIs. The pre/post subgroup difference (38.1% vs. 63.0%) is descriptive only; subgroup sizes are small and confidence intervals are wide and overlapping, and this analysis cannot separate any true change in screening performance from the confounding effects described in manuscript Sections 3.1 and 4.3.
